# Nanoliposomal Formulation of *Gynura procumbens* Leaf Extract Potentiates Hepatorenal Protection in Cisplatin‐Induced Rats

**DOI:** 10.1002/fsn3.71035

**Published:** 2025-09-28

**Authors:** Leon Bhowmik, Ashikur Rahman, Madhobi Karmakar, S. M. M. Sharif Nowaz Antu, Zahidul Islam Zahid, Madhabi Lata Shuma, Shazid Md. Sharker, Hasan Mahmud Reza, Shimul Halder, Manik Chandra Shill

**Affiliations:** ^1^ Department of Pharmaceutical Sciences North South University Dhaka Bangladesh; ^2^ Department of Pharmacy, School of Pharmacy and Public Health Independent University Bangladesh Dhaka Bangladesh; ^3^ Department of Pharmaceutical Technology, Faculty of Pharmacy University of Dhaka Dhaka Bangladesh

**Keywords:** cisplatin, *Gynura procumbens*, hepatoprotective, liposomal drug delivery system, nephroprotective

## Abstract

*Gynura procumbens*, commonly known as longevity spinach, is traditionally used in Southeast Asia for its hepatoprotective, anti‐inflammatory, antihypertensive, and antihyperglycemic properties. This study aimed to enhance the hepatorenal protective effects of *G. procumbens* leaf extract (GLE) by incorporating it into a nanoliposomal drug delivery system (LIP), thereby improving its dispersibility/solubility and therapeutic efficacy. The resulting nano‐formulation, LIP–GLE, produced micelles with an average size of 112 ± 2.6 nm, showing a 6.6‐fold and 4.6‐fold improvement in dispersibility in water and simulated gastric fluid, respectively, compared to GLE. In a rat model of cisplatin‐induced acute hepatorenal injury (7.5 mg/kg, *i.p*.), oral administration of LIP–GLE (75 mg GLE/kg) significantly improved liver and kidney function, as indicated by reduced serum levels of ALT, AST, ALP, BUN, and creatinine. Histopathological investigations further confirmed reduced tissue damage in the liver and kidneys. Additionally, LIP–GLE enhanced antioxidant enzyme activities (SOD, GSH) and reduced oxidative stress markers (NO, AOPP), indicating strong protective effects against cisplatin‐induced oxidative injury. These findings demonstrate that liposomal encapsulation significantly enhances the bioavailability and therapeutic potential of GLE, making it a promising approach for enhancing the nutraceutical potential of *G. procumbens*.

## Graphical abstract

*Gynura procumbens* is a medicinal plant traditionally used for its anti‐inflammatory and antioxidant properties. However, its therapeutic efficacy is often limited by poor solubility and bioavailability. In this study, we aimed to enhance its pharmacological potential by incorporating the leaf extract into a nanoliposomal delivery system. We evaluated the protective effects of this formulation in a rat model of cisplatin‐induced hepatorenal toxicity. Biochemical, histopathological, and oxidative stress markers were assessed to determine the extent of organ protection. The nanoliposomal formulation markedly improved the bioactivity of *G. procumbens*, as evidenced by significant reductions in serum creatinine, ALT, AST, and ALP levels, along with preservation of normal tissue architecture in the liver and kidneys. The enhanced effect is attributed to improved cellular uptake and sustained release of bioactive compounds due to liposomal encapsulation. This study supports the traditional use of GLE in mitigating organ damage and demonstrates the value of nanoliposomal systems in optimizing the delivery and therapeutic efficiency of herbal medicines.

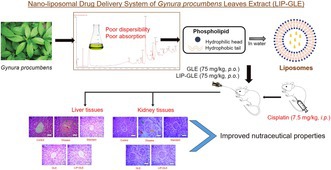

## 1 Introduction

Since ancient times, plants have been integral to human life, providing food, clothing, shelter, and medicinal remedies. Many ancient cultures recognized the therapeutic value of herbal medicine, which remains an important alternative treatment option. Plants and herbs have been used to manage various ailments, including urinary disorders, diabetes, asthma, stroke, hypertension, diarrhea, and wounds (Drews 2000; Newman and Cragg 2020). According to the World Health Organization (WHO), about 70% of the global population uses traditional or folk medicine due to its accessibility, efficacy, and affordability (Hamilton 2004; Rates 2001). Conventional medicine remains crucial for treating chronic and life‐threatening diseases, such as metabolic, endocrine, respiratory, neurological disorders and cancer (Amrati et al. 2021; Ernst 2000; Puri et al. 2022). However, interest in complementary and alternative therapies, particularly herbal remedies, continues to grow.

*Gynura procumbens*, a prominent medicinal plant in Bangladesh, belongs to the Asteraceae family and possesses a multitude of therapeutic advantages, including antihypertensive, antihyperglycemic, cardioprotective, fertility‐enhancing, antioxidant, antimicrobial, organ‐protective, anti‐inflammatory, and anticancer properties (Jobaer et al. 2023; Tan et al. 2016) (Hew et al. 2013).

Despite its diverse phytochemical profile and therapeutic potential, the clinical and nutraceutical application of *G. procumbens* leaf extract (GLE) faces major delivery challenges. Its bioactive compounds have poor water solubility, low stability in the gastrointestinal tract, and limited oral absorption (Shill et al. 2025; H.‐L. Tan et al. 2016). During digestion, these compounds may degrade, and low absorption further reduces therapeutic efficacy (Wardana et al. 2023). To fully unlock GLE's potential, new delivery methods are needed to improve its solubility, stability, and availability in the body.

Nano‐drug delivery systems offer a promising solution to these issues, providing an innovative approach to overcoming the limitations of traditional plant extract formulations (Bonifácio et al. 2014). Various types of nanocarriers, including nanoparticles, nanoemulsions, solid lipid nanoparticles, and liposomes, have been explored to enhance the delivery of poorly soluble bioactive compounds (Ibrahim et al. 2020). Among these, liposomal systems stand out for their unique advantages, particularly in encapsulating plant extracts (Coimbra et al. 2011). Liposomes, in particular, offer advantages for plant extract delivery due to their biocompatibility, ability to protect phytochemicals from enzymatic degradation, and capacity to improve permeability and sustain release (Allen and Cullis 2013; Coimbra et al. 2011; Nsairat et al. 2022). Despite the extensive use of nanocarrier systems in synthetic compounds, the application of liposomal technology in bioactive extracts like GLE has not been thoroughly explored. This research aimed to study the protective properties of LIP–GLE in mitigating cisplatin‐induced damage to the liver and kidneys in rats, delving into its potential for advancing the nutraceutical and therapeutic applications of GLE through nanotechnology‐based delivery systems, filling an important gap in plant extract‐based drug delivery research.

## 2 Materials and Methods

### 2.1 Chemicals and Reagents

Phosphatidylcholine, derived from soy lecithin (Cat. No. L0023), and cholesterol (Cat. No. C0318) was procured from Tokyo Chemical Industries (Tokyo, Japan). Meanwhile, silymarin (Cat. No. S0292) and cisplatin (Cat. No. P4394) were acquired from Sigma‐Aldrich (St. Louis, MO, USA). All other chemicals, reagents, and solvents used in the study were of analytical grade and sourced from acceptable commercial suppliers.

### 2.2 Collection, Identification, and Extraction of Plant Materials

After gathering from Natore, Bangladesh, *G. procumbens* leaves were verified at the National Herbarium in Mirpur, where a voucher specimen was placed (DACB accession number: 45273). After thorough cleaning and air‐drying in the shade, the leaves were finely ground and extracted with ethanol. Following a previously established protocol, the resulting filtrate was concentrated using a rotary evaporator to obtain the crude extract (Chandra Shill, All Rakib, et al. 2024; Shill et al. 2025).

### 2.3 GC–MS Analysis for GLE Composition

Gas chromatography–mass spectrometry (GC–MS) was used to analyze the phytochemical composition of GLE. The analysis was conducted using a Clarus 690 gas chromatograph (PerkinElmer, CA, USA) fitted with an HP‐5MS capillary column (30 m × 0.25 mm, 0.25 μm film thickness), coupled to a Clarus SQ 8 C mass spectrometer (PerkinElmer, CA, USA). A 1 μL sample of GLE was injected in splitless mode, with high‐purity helium (99.999%) serving as the carrier gas at a constant flow rate of 1 mL/min over a 60 min run. The analysis was performed in electron ionization (EI) mode at 70 eV. The injector temperature was set to 280°C, and the oven temperature was programmed to start at 60°C (no hold), then ramped up at 4°C per min to 240°C, where it was held for 15 min (Zilani et al. 2021). Chemical compounds were identified by comparing their mass spectra with entries in the National Institute of Standards and Technology (NIST) database (Stein et al. 2011).

### 2.4 Preparation of LIP–GLE

In this study, LIP–GLE was prepared using the thin film hydration method, as described previously (Zhang 2017). Briefly, GLE‐lecithin and cholesterol were mixed in a 1:1 ratio and dissolved in ethanol. This solution was then evaporated using a rotary evaporator to form a thin, dry film on the inner surface of a round‐bottom flask. The film was then rehydrated with 10 mL of water and subjected to sonication. After 30–40 min of sonication at approximately 50°C, a milky‐greenish liposomal formulation (LIP–GLE) was obtained. This process yielded a more uniform preparation, consisting primarily of multilamellar vesicles (MLVs) (Xiang and Cao 2017).

### 2.5 Dynamic Light Scattering (DLS)

The particle size distribution of LIP–GLE was evaluated using dynamic light scattering (DLS) with an SZ‐100 nanoparticle analyzer (Horiba, Japan). For the analysis, the LIP–GLE sample was diluted in Milli‐Q water to maintain a GLE concentration of 10 μg/mL. Measurements were carried out at 25°C with a detection angle of 90°. To assess colloidal stability, both the zeta potential and the polydispersity index (PDI) were measured. All tests were conducted in triplicate to ensure accuracy and reproducibility.

### 2.6 Transmission Electron Microscopy (TEM)

The surface morphology of the LIP–GLE was examined using TEM to observe droplet structure and size. The formulation was diluted with Milli‐Q water (0.5%, w/v) and applied to a 300‐mesh copper grid coated with carbon. Negative staining was performed using a 2% (w/v) phosphotungstic acid (PTA) solution and gentle rinsing with Milli‐Q water to remove excess stain. The grid was then oven‐dried under vacuum to eliminate moisture. TEM analysis was conducted using a Talos F200X microscope (Thermo Fisher, Waltham, MA, USA) operated at 200 kV. ImageJ software (NIH, Bethesda, MD, USA) assessed and confirmed droplet size and morphology.

### 2.7 Interaction of GLE With Polymers

The compatibility and potential interactions among the components of the LIP–GLE were evaluated using Attenuated Total Reflectance‐Fourier Transform Infrared (ATR‐FTIR) spectroscopy to ensure the stability and integrity of the formulation. ATR–FTIR analysis was conducted using a Shimadzu IR‐Prestige‐21 FTIR spectrometer (Tokyo, Japan) equipped with a horizontal Golden Gate MKII single‐reflection ATR system (Specac, Kent, UK). Each individual sample, including GLE, lecithin, cholesterol, and the LIP–GLE, was carefully placed on the sample platform to avoid contamination and interference. Spectra were recorded within the wavenumber range of 600–4000 cm^−1^ at a resolution of 4 cm^−1^, with 64 scans averaged per spectrum to improve signal‐to‐noise ratio and accuracy. The resulting spectra were analyzed using IRsolution 1.30 software (Shimadzu, Tokyo, Japan), with baseline correction and normalization applied to ensure accurate comparison between samples.

### 2.8 In Vitro Dissolution/Dispersion Tests

To evaluate the dispersibility of GLE, 1 g of the sample was tested using an in vitro dissolution/dispersion setup. The experiment was conducted with a USP Type‐II dissolution apparatus, using two different media: distilled water and a pH 1.2 solution (0.1 N HCl). The test was performed in 900 mL of medium maintained at 37°C ± 0.5°C, with constant stirring at 50 rpm for 60 min. Samples were collected from the center of the vessel at 5, 15, 30, 45, and 60 min intervals using a micropipette. After each sample was taken, the same volume of fresh medium was added to maintain consistency. The collected samples were then centrifuged at 10,000 × *g* for 10 min and filtered through a 0.45 μm membrane filter (Millex LG, Millipore, Billerica, MA). The resulting filtrates were diluted with 50% methanol, and the concentration of dispersed or dissolved GLE was measured using a UV spectrophotometer at 254 nm (Mohammed et al. 2019).

### 2.9 Animals

Healthy male Wistar albino rats, weighing between 250 and 300 g, were obtained from North South University, Dhaka, Bangladesh, for the experimental study. The animals were housed in pairs in standard laboratory cages under controlled conditions, including a 12‐h light/dark cycle, a temperature of 24°C ± 1°C, and a relative humidity of 55% ± 5%. They had free access to standard laboratory chow and water. Before oral administration of the GLE samples, all rats were fasted for at least 12 h to standardize absorption conditions, during which food and water were withheld. All animal procedures were carried out following ethical guidelines approved by the Institutional Animal Care and Ethical Committee of North South University (Approval No. 2021/OR‐NSU/IACUC/1102), following international standards set by the Council for International Organizations of Medical Sciences (CIOMS/ICLAS), the Nuffield Council on Bioethics (NCB), and the International Council for Laboratory Animal Science (Turner et al. 2015).

### 2.10 Experimental Rat Model of Kidney and Liver Injury

The protective effects of GLE samples against cisplatin‐induced liver and kidney damage were assessed using a well‐established rat model of acute hepatorenal toxicity. To induce organ injury, a single intraperitoneal dose of cisplatin (7.5 mg/kg in saline) was administered on the seventh day of the experiment (Aboraya et al. 2022; Anwer et al. 2023). Rats were randomly assigned to five groups, with six rats in each group. The study followed ethical guidelines set by the Nuffield Council on Bioethics (NCB) and CIOMS/ICLAS, and group sizes ranging from 4 to 6 animals were used based on statistical power calculations aimed at minimizing animal use (Arifin and Zahiruddin 2017). Group I served as the control group (received only saline), Group II was the disease model group (received cisplatin only), Group III was the standard treatment group (received cisplatin and silymarin [100 mg/kg, *p.o*.]), Group IV received cisplatin and GLE (75 mg/kg, *p.o*.), and Group V received cisplatin and LIP–GLE (75 mg/kg, *p.o*.). Both GLE and LIP–GLE were administered orally to evaluate their nephroprotective and hepatoprotective effects, with dosages determined from prior experimental data (Chandra Shill, All Rakib, et al. 2024; Shill et al. 2025). Following 10 days' treatment, rats were anesthetized with ketamine (100 mg/kg, *i.p*.), blood samples were obtained, centrifuged at 10,000 × *g* for 10 min, and serum was stored at −80°C until analysis. After blood collection, the rats were euthanized humanely using a high dose of ketamine (300 mg/kg, *i.p*.) to collect kidney and liver tissues. Liver and kidney tissues were carefully excised, rinsed with ice‐cold saline to remove blood residues, blotted dry with sterile filter paper, and preserved in 10% neutral buffered formalin for histopathological evaluation. All experimental procedures were carried out in accordance with ethical standards approved by the Institutional Animal Care and Ethical Committee of North South University (Approval No. 2021/OR‐NSU/IACUC/1102), with strict adherence to humane endpoints to prevent undue suffering to the animals.

### 2.11 Evaluation of Serum Biomarkers

Serum biomarkers were evaluated to assess nephrotoxicity and hepatotoxicity in rats. Blood urea nitrogen (BUN; Cat. No. E‐BC‐K183‐S.50) and creatinine (Cat. No. Cay500701‐96) were measured using diagnostic kits from HUMAN GmbH, Wiesbaden, Hesse, Germany, to determine renal function. Measurement of hepatic biomarkers was carried out using commercially available reagent kits from Fortress Diagnostics Limited (Northern Ireland, United Kingdom): alanine aminotransferase (ALT) (GPT, IFCC, liquid‐stable; Cat. No. BXC0213A), aspartate aminotransferase (AST) (GOT, IFCC, liquid‐stable; Cat. No. BXC0203A), and alkaline phosphatase (ALP) (L.S, AMP, IFCC; Cat. No. BXC0184C). All biomarker levels were determined spectrophotometrically at a wavelength of 340 nm, following the respective manufacturers' protocols (Veskoukis et al. 2018). These biochemical parameters served as key indicators of organ function to evaluate the protective effects of GLE and LIP–GLE against cisplatin‐induced toxicity.

### 2.12 Oxidative Stress and Antioxidant Activity Measurement

The collected samples were homogenized and sonicated to assess oxidative stress and antioxidant activity in liver and kidney tissues, then centrifuged at 10,000 × *g* for 15 min at 4°C. The resulting supernatants were carefully collected and stored for subsequent biochemical analysis. Oxidative stress markers, including nitric oxide (NO) and advanced oxidative protein products (AOPP), were measured in the tissue homogenates using established methods (Chandra Shill, El‐Nashar, et al. 2024; Shill et al. 2021) to evaluate the extent of damage caused by cisplatin. In addition, the activity levels of key antioxidant enzymes, superoxide dismutase (SOD) and glutathione (GSH), were determined in both liver and kidney samples, following standard protocols (Lamia et al. 2021). These antioxidant markers provide insight into the tissue's defense mechanisms against free radical damage and lipid peroxidation. By examining both oxidative damage and antioxidant responses, the study aimed to evaluate the protective effects of GLE and LIP–GLE in mitigating oxidative stress and preserving liver and kidney function.

### 2.13 Histopathological Examination

Histopathological analysis of liver and kidney tissues was carried out using a modified version of the protocol described by Quaresma et al. (2005). The tissues were first fixed in 10% neutral buffered formalin, then rinsed thrice with phosphate‐buffered saline (PBS, pH 7.4). To ensure cryoprotection, the samples were placed in a solution of 30% sucrose and 0.1% sodium azide at 4°C for 24 h. Afterward, the tissues were embedded in paraffin wax and sectioned into 5 μm slices using a rotary microtome. The sections were stained with hematoxylin and eosin (H&E) for evaluating tissue structure and inflammatory cell infiltration and with Sirius red to assess the presence and extent of fibrosis. Histological features of hepatic and renal inflammation, structural alterations, and fibrotic changes were observed and documented using a Zeiss Axioscope 40‐X light microscope (Germany). Quantifying the density of inflammation and fibrosis in both liver and kidney tissues was performed using ImageJ software (NIH, USA), applying a standardized analysis protocol to evaluate the severity of tissue damage (Jensen 2013; Shill et al. 2021).

### 2.14 Data Analysis

Data are expressed as mean ± standard deviation (SD) or mean ± standard error of the mean (SEM). Statistical analysis was performed using one‐way ANOVA in GraphPad Prism‐8 software (San Diego, CA, USA). Before performing one‐way ANOVA, the underlying assumptions were tested. Independence of observations was ensured by the experimental design, as each subject was assigned to only one treatment group. The normality of residuals was assessed using the Shapiro–Wilk test, and all data sets showed no significant deviation from normality (*p* > 0.05). Homogeneity of variances among groups was evaluated using Levene's test, which confirmed equal variances (*p* > 0.05). These conditions justified the use of one‐way ANOVA for group comparisons. Differences between groups were considered statistically significant when the *p*‐value was less than or equal to 0.05.

## 3 Results and Discussion

### 3.1 GC–MS Study

**FIGURE 1:**
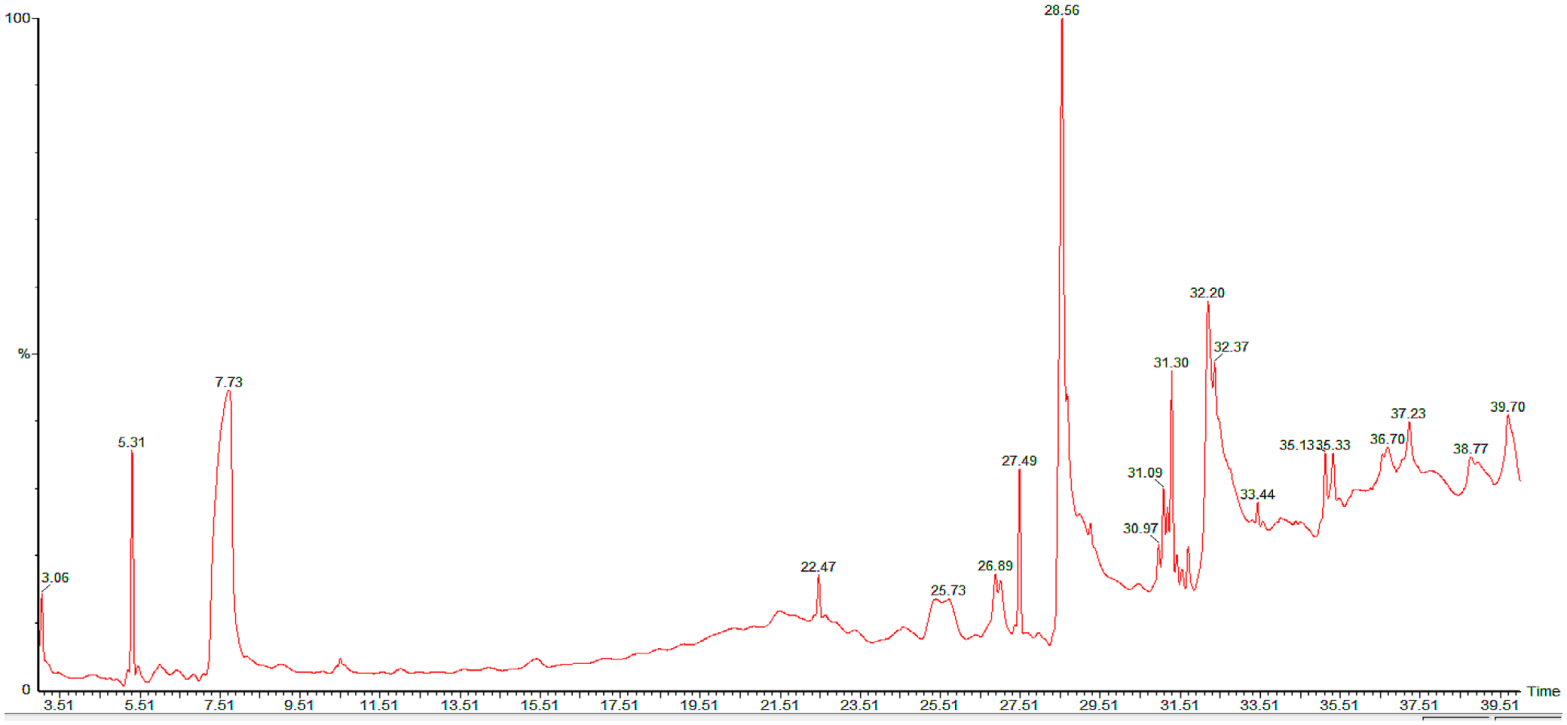
Gas chromatography–mass spectrometric (GC–MS) chromatogram of GLE.

The GC–MS spectrum presented in Figure 1 illustrates the phytochemicals present in GLE. The composition of bioactive compounds obtained from GLE is presented in Table 1, which includes their retention time (RT), molecular formula, molecular weight, and peak area (%). The most abundant one is n‐hexadecanoic acid, followed by 2‐marcaptopropanoic acid and methyl 2‐hydroxy‐heptadecanoate. N‐Hexadecanoic acid, 2‐marcaptopropanoic acid, and methyl 2‐hydroxy‐heptadecanoate are among the significant components that GC–MS data indicate were detected in the extract of GLE, among others (Figure 1 and Table 1). According to prior studies, n‐hexadecanoic acid has various pharmacological effects, including anticancer, hepatoprotective, anti‐inflammatory, and antioxidant activities. A literature review revealed that GLE possesses potent anti‐inflammatory and antioxidant properties, which may be related to the presence of methyl 2‐hydroxy‐heptadecanoate (Kim et al. 2021; J. N. Tan et al. 2020). GLE has limited intestinal absorption, is unstable at acidic pH levels, and is weakly soluble in water. Hence, a liposomal drug delivery method was used in this study because a nanocarrier‐based drug delivery system might be able to enhance the solubility and stability of the components found in the GLE to enhance solubility and bioavailability as well as provide superior physicochemical stability.

**TABLE 1:** List of identified compounds in the crude extract of *Gynura procumbens* through GC–MS analysis.

Retention time (min)	Chemical name	% Peak area
7.71	2‐Mercaptopropanoic acid	15.04
22.47	Di‐epi‐1,10‐cubenol	4.65
27.48	Tetradecanoic acid, 10,13‐dimethyl‐, methyl ester	1.79
28.55	N‐hexadecanoic acid	19.39
30.45	Phthalic acid, isobutyl 2‐methylpent‐3‐yl ester	0.15
31.10	Cyclopropaneundecanal, 2‐nonyl—	1.14
31.29	Heptadecanoic acid, 16‐methyl‐, methyl ester	2.23
32.21	Methyl 2‐hydroxy‐heptadecanoate	14.72
35.14	Glycidyl palmitate	0.63

### 3.2 Physicochemical Characterizations of LIP–GLE

**FIGURE 2:**
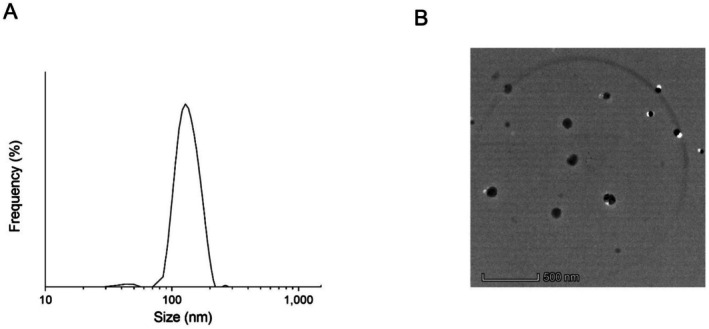
Particle size distribution of GLE samples dispersed in water. (A) DLS analysis determined the size distribution in distilled water. (B) TEM image of LIP–GLE dispersed in distilled water. Bar represents 500 nm.

The LIP–GLE was characterized for size distribution, PDI, zeta potential, and morphology using DLS and TEM techniques. Liposomes with a narrow particle size serve as passive targeting methods to concentrate at sites, primarily attributed to the enhanced permeability and retention effect. The DLS analysis revealed that the GLE did not exhibit a defined nanoparticulate nature, with irregular and larger particle distribution, indicating poor dispersibility and potential limitations in bioavailability (data not shown). In contrast, the LIP–GLE exhibited a consistent particle size distribution, with an average hydrodynamic diameter of around 112 ± 2.6 nm and a low PDI of 0.231 ± 0.04, indicating favorable homogeneity and stability. The zeta potential was measured at −16.1 ± 3.1 mV, suggesting high colloidal stability due to strong electrostatic repulsion, which prevents aggregation and enhances shelf life. TEM imaging confirmed the liposome's spherical morphology and well‐defined nanostructure (Figure 2B), consistent with the DLS data. The small particle size and stable zeta potential likely facilitated efficient penetration into hepatocytes and renal cells, mitigating oxidative stress and inflammation induced by cisplatin. The results highlight the promise of LIP–GLE as an innovative approach to minimize the hepatorenal toxicity induced by cisplatin.

### 3.3 In Vitro Dispersion Study

**FIGURE 3:**
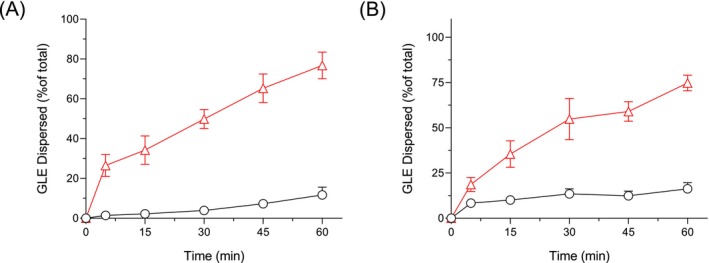
Dissolution/dispersion tests of GLE samples. (A) Dissolution/dispersion tests in distilled water; (B) dissolution/dispersion tests under acidic conditions (0.1 N HCl, pH 1.2). ●, GLE and △, LIP–GLE. Each bar represents the mean ± SD of 3 independent experiments.

The in vitro dispersion study was conducted to assess and compare the dispersibility of the GLE samples in distilled water and 0.1 N HCl over 60 min (Figure 3). In distilled water, GLE exhibited a relatively poor dispersion profile, with only about 11.7% of the extract dispersed at the end of 60 min, likely due to its hydrophobic phytoconstituents and larger particle aggregates. Conversely, LIP–GLE showed a significantly higher dispersion rate, reaching approximately 76.7% within the same duration, highlighting enhanced solubility of its phytoconstituents and interaction with the aqueous medium. A similar trend was observed in simulated gastric media (0.1 N HCl), where GLE dispersed up to only 16.3%, whereas LIP–GLE achieved over 74.7% dispersion, indicating superior performance even under acidic conditions. These improvements in dispersion correlate strongly with the droplet size of the LIP–GLE (Figure 2). The small droplet size provides a larger surface area‐to‐volume ratio, facilitating more efficient wetting and solubilization of the extract in both neutral and acidic media. Furthermore, the liposomal system's phospholipid bilayers improve the encapsulated extract's stability and dispersibility by creating a protective and hydrophilic interface around the hydrophobic components. This improved dispersion indicates superior solubility and suggests enhanced gastrointestinal absorption, which is crucial for the systemic bioavailability of the active compounds. The limited dispersibility of the GLE could hinder its therapeutic effectiveness, resulting in incomplete solubilization and variable absorption. Overall, the in vitro dispersion data, supported by particle size analysis, clearly indicate the superiority of the liposomal formulation in enhancing the solubility, stability, and potential bioavailability of GLE, ultimately contributing to its enhanced protective effects against hepatorenal injury.

### 3.4 Drug–Polymer Interactions

**FIGURE 4:**
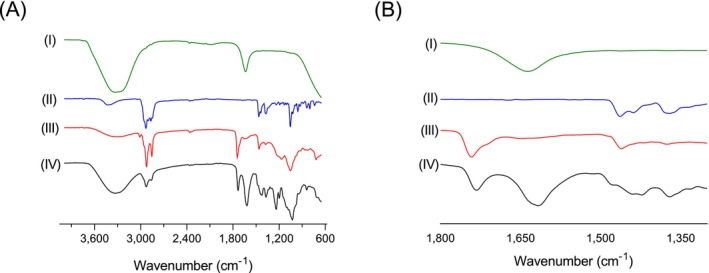
ATR‐FTIR spectroscopic analysis of GLE samples in the spectral region from (A) 4000–600 cm^−1^ and (B) 1800–1300 cm^−1^. (I) LIP–GLE, (II) cholesterol, (III) lecithin, and (IV) GLE.

FTIR analysis was conducted to evaluate the molecular interactions among the components of LIP–GLE, as illustrated in Figure 4. The FTIR spectrum of GLE exhibited significant peaks at 1370 and 1167 cm^−1^, which are associated with C–H bending and C–O stretching vibrations, suggesting the presence of alcohol groups. Prominent absorption bands observed at 2923 and 2853.6 cm^−1^ correspond to C–H stretching in aliphatic methyl and isopropyl groups. A sharp band at 1743.7 cm^−1^ confirmed carbonyl (C=O) stretching, indicative of ketones or esters. Additional peaks at 1463.8 and 1377.5 cm^−1^ reflected scissoring vibrations of C–H bonds and methyl group activity, respectively. Lecithin, a phospholipid, exhibited its characteristic ester carbonyl peak near 1742.5 cm^−1^, while cholesterol demonstrated distinct absorption bands around 3432.08 cm^−1^ due to –OH stretching and 1463.53 cm^−1^ for C–H bending. Its lipid backbone was also evident from C–H stretching and bending at 2899.99 and 1434.92 cm^−1^. Upon formulation of LIP–GLE, several changes were observed in the FTIR spectra: key GLE peaks appeared broadened or slightly shifted, particularly within the 3300–3200 cm^−1^ region, indicating altered hydrogen bonding, and in the 1700–1500 cm^−1^ range (Figure 4B), reflecting non‐covalent interactions with lipid components. The lecithin and cholesterol peaks also showed shifts and overlaps, confirming successful interaction. These spectral modifications suggest effective physical encapsulation of GLE in the liposomal bilayer through hydrogen bonding, van der Waals interactions, and other weak forces, rather than covalent bond formation. Notably, no new peaks or loss of essential functional group signals were detected, ruling out chemical incompatibility and confirming that the extract retained its chemical identity. This supports the hypothesis that GLE was physically entrapped within the lipid matrix, maintaining its bioactive properties. These molecular interactions contribute to the nanoliposome's structural stability, enhance solubility, and improve the permeability and sustained release profile of GLE. Consequently, the FTIR findings affirm the compatibility and structural integrity of the formulation and highlight that lipid‐based encapsulation synergistically enhances the therapeutic potential of GLE by safeguarding it from degradation and optimizing its pharmacological performance.

### 3.5 General Conditions of the Treated Animals

During the experimental period, all animals were monitored daily for clinical signs, symptoms, and mortality. No deaths were observed in any group. Rats exhibited progressive dehydration, weight loss, anemia, reduced activity, and gastrointestinal distress within 2 days of receiving cisplatin (7.5 mg/kg) (Perše and Veceric‐Haler 2018). Rats in the cisplatin‐treated group showed signs of reduced activity, mild piloerection, and decreased food intake. Body weight measurements revealed that cisplatin administration significantly reduced weight gain compared to the control group (*p* < 0.05).

### 3.6 Effect of GLE Samples on Liver Functions

**FIGURE 5:**
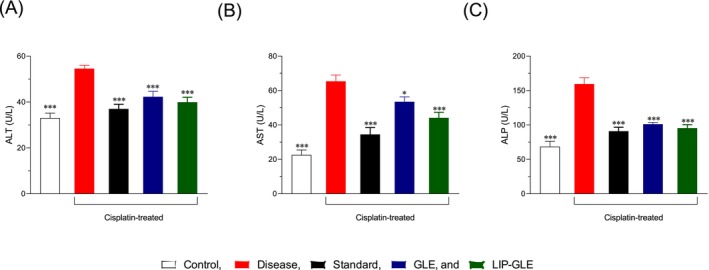
Effect of GLE samples on hepatic markers in the rat liver: (A) alanine aminotransferase (ALT), (B) aspartate aminotransferase (AST), and (C) alkaline phosphatase (ALP) levels. The data were expressed as Mean ± SEM; (*n* = 6), where n is the number of rats. *p < 0.05; ***p < 0.001; with respect to cisplatin‐treated rats.

The hepatoprotective potential of GLE samples was evaluated by assessing liver function biomarkers: ALT, AST, and ALP presented in Figure 5. In the control group, baseline values were ALT: 33.0 ± 1.90 U/L, AST: 22.5 ± 2.59 U/L, and ALP: 68.5 ± 6.78 U/L, respectively. The diseased group showed significantly elevated levels (ALT: 54.58 ± 1.40 U/L, AST: 65.4 ± 3.59 U/L, ALP: 159.5 ± 9.06 U/L, *p* < 0.001 vs. control), reflecting cisplatin‐induced renal damage. Treatment with the standard hepatoprotective agent silymarin significantly ameliorated these effects, with values recorded as ALT (37.07 ± 1.98 U/L), AST (34.5 ± 3.94 U/L), and ALP (91.0 ± 5.69 U/L) (*p* < 0.001 vs. disease group). The GLE‐treated group showed moderate protection: ALT (42.3 ± 2.34 U/L), AST (53.5 ± 2.86 U/L), and ALP (101.1 ± 2.65 U/L) (*p* < 0.001 vs. disease group), highlighting the beneficial role of its bioactivity but limited efficacy. The LIP–GLE group exhibited superior restoration, with ALT (39.9 ± 2.13 U/L), AST (44.1 ± 3.26 U/L), and ALP (95.5 ± 4.94 U/L) (*p* < 0.001 vs. disease group), nearly restoring enzyme levels to those of the control group. Cisplatin administration leads to oxidative stress, mitochondrial dysfunction, and disruption of hepatocyte membranes, resulting in the leakage of these enzymes into circulation (Haimoud et al. 2023). Elevated levels of ALT and AST reflect hepatocellular injury, while increased ALP indicates biliary tract dysfunction. In this study, LIP–GLE showed a significant reduction in all three enzymes compared to the disease group, indicating its effectiveness in stabilizing hepatocyte membranes and improving liver function. The superior efficacy of LIP–GLE over GLE can be attributed to the enhanced bioavailability and better cellular penetration provided by the liposomal system, which facilitates more efficient delivery of antioxidant and anti‐inflammatory compounds (Sajid et al. 2018). These compounds likely helped reduce oxidative stress, protect mitochondrial function, and inhibit inflammatory pathways, leading to less enzyme leakage. The normalization of ALT, AST, and ALP levels in the LIP–GLE group suggests that it can effectively counteract cisplatin‐induced liver injury by stabilizing cell membranes, restoring antioxidant defenses, and preventing hepatocellular apoptosis, highlighting its potential as a therapeutic agent.

### 3.7 Effect of GLE Samples on Kidney Functions

**FIGURE 6:**
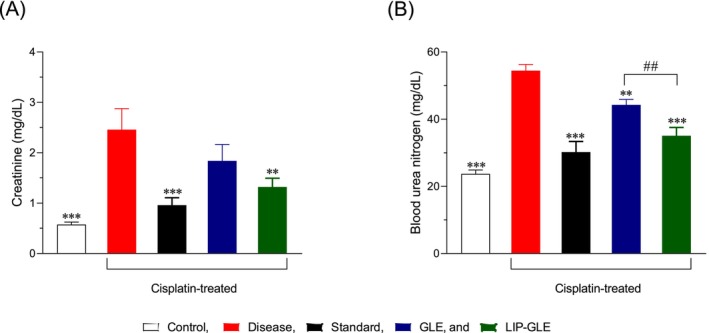
Nephroprotective potential of GLE and LIP–GLE in a rat nephropathy model induced by cisplatin. (A) Serum creatinine and (B) BUN levels in rats with orally administered GLE and LIP–GLE. **p < 0.01; ***p < 0.001; with respect to cisplatin‐treated rats; ^##^ *p* < 0.05, GLE vs. LIP–GLE. Data represents Mean ± SEM; (*n* = 6).

The impact of GLE samples on cisplatin‐induced renal dysfunction was assessed through the quantification of creatinine and BUN and is presented in Figure 6. In the control group, creatinine and BUN levels remained within normal physiological ranges (0.57 ± 0.04 mg/dL and 23.7 ± 1.03 mg/dL, respectively), indicating optimal renal function. However, the disease group treated with cisplatin alone showed a significant elevation (*p* < 0.001 vs. control) in creatinine (2.46 ± 0.42 mg/dL) and BUN (54.4 ± 1.85 mg/dL), reflecting marked nephrotoxicity and impaired glomerular filtration. Treatment with the standard nephroprotective agent silymarin resulted in a considerable decrease in these markers (creatinine: 0.96 ± 0.15 mg/dL; BUN: 30.2 ± 3.16 mg/dL, *p* < 0.001 vs. disease), indicating partial protection. GLE administration also significantly reduced (*p* < 0.001 vs. disease), with creatinine and BUN levels recorded at 1.84 ± 0.32 mg/dL and 44.2 ± 1.67 mg/dL, respectively, suggesting moderate renoprotective potential. In contrast, the LIP–GLE group demonstrated superior restoration (creatinine: 1.32 ± 0.17 mg/dL, BUN: 35.1 ± 2.48 mg/dL, *p* < 0.001 vs. disease), surpassing GLE. This enhanced therapeutic effect is attributed to improved solubility, cellular permeability, and sustained release of bioactive compounds provided by the liposomal system, which ensures better pharmacokinetic and pharmacodynamic outcomes. LIP–GLE likely amplified antioxidant and anti‐inflammatory actions, countering cisplatin‐induced oxidative stress and tubular damage. Thus, LIP–GLE's superior performance over GLE suggests it is a promising therapeutic strategy for mitigating cisplatin‐induced renal injury, warranting further clinical exploration.

### 3.8 Effect of GLE Samples on Oxidative Stress in Hepatorenal Protection

**FIGURE 7:**
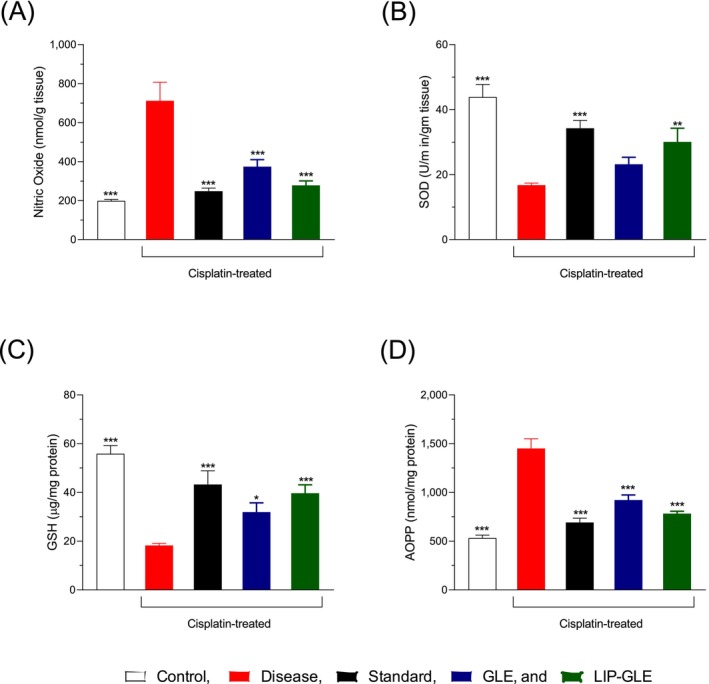
Effect of LIP–GLE on oxidative stress and antioxidant enzymes in experimental rats induced by cisplatin in liver (A) NO, (B) SOD, (C) GSH, and (D) AOPP. Data are expressed as means ± SEM; *p ≤ 0.05; **p ≤ 0.01; ***p ≤ 0.001 versus control, disease, or treatment groups (*n* = 6).

The effects of GLE on oxidative stress were evaluated by measuring NO and AOPP levels in cisplatin‐induced hepatorenal toxicity, presented in Figure 7. In the control group, NO and AOPP levels remained within normal limits, indicating balanced redox homeostasis in hepatic and renal tissues. In contrast, the disease group exhibited a significant elevation (*p* < 0.001 vs. control) in both NO and AOPP levels, reflecting severe oxidative stress triggered by cisplatin administration, which promotes reactive oxygen species formation and protein oxidation, leading to cellular and tissue damage. Treatment with silymarin resulted in a moderate reduction in oxidative markers, suggesting partial antioxidant protection. Similarly, administering GLE led to a significant, though somewhat less pronounced, decrease in NO and AOPP levels (Figure 7) compared to the disease group, indicating its potential antioxidant effects. However, the LIP–GLE group showed a substantial reduction in NO and AOPP concentrations, bringing them closer to control group levels and highlighting its superior antioxidative effectiveness. This enhanced efficacy of LIP–GLE can be attributed to its nanoliposomal encapsulation, which improves the solubility and targeted delivery of the bioactive components in the extract, allowing for more efficient free radical scavenging and reduced oxidative damage in both liver and kidney tissues (Ghosh et al. 2023). These findings are consistent with the improved biomarker levels seen in the LIP–GLE group, further emphasizing the crucial role of oxidative stress in cisplatin‐induced organ damage. The reduction in NO and AOPP levels in the LIP–GLE group underscores its potential as a protective agent against oxidative stress‐related damage to the liver and kidneys.

### 3.9 Effect of GLE Samples on Antioxidant Enzymes

**FIGURE 8:**
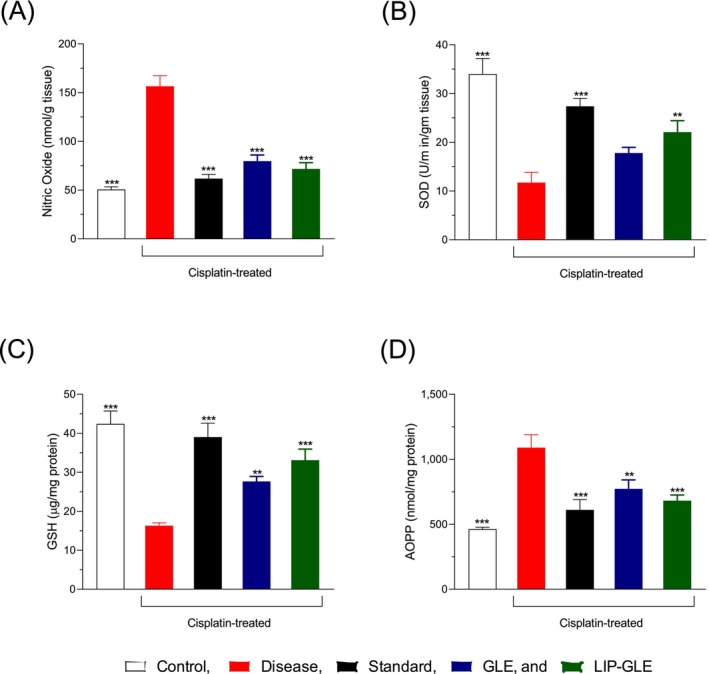
Effect of LIP–GLE on oxidative stress and antioxidant enzymes in experimental rats induced by cisplatin in Kidney (A) NO (B) SOD (C) GSH and (D) AOPP. Data are expressed as means ± SEM; **p ≤ 0.01; ***p ≤ 0.001 versus control or disease or treatment groups (*n* = 6).

The assessment of antioxidant defense mechanisms in the context of cisplatin‐induced liver and kidney damage involved measuring GSH and SOD levels, as shown in Figure 8. In the control group, both GSH and SOD levels remained within normal physiological ranges, indicating that the liver and kidneys were maintaining their antioxidant defense systems. However, a significant decrease (*p* < 0.001 vs. control) in both GSH and SOD levels was observed in the disease group treated with cisplatin alone, reflecting severe oxidative stress and a reduction in intrinsic antioxidant enzymes caused by the accumulation of reactive oxygen species (ROS) and resulting tissue damage. Treatment with silymarin notably improved GSH and SOD levels compared to the disease group, highlighting its known antioxidant properties. GLE treatment also boosted antioxidant enzyme levels, though to a lesser extent than silymarin, suggesting a moderate ability to counteract oxidative stress. The LIP–GLE group showed a marked increase in both GSH and SOD levels, nearly returning them to the control group levels, indicating enhanced antioxidant protection. This increase in antioxidant enzyme activity is linked to the improved performance and targeted delivery of the extract's bioactive components through the nanoliposomal formulation, which enhances cellular uptake and prolongs antioxidant effects (Abd Rashid et al. 2021). These results align with the reduced oxidative stress markers and improved biomarkers in the liver and kidney tissues. The ability of LIP–GLE to restore antioxidant enzyme levels underscores the importance of maintaining redox balance to protect against cisplatin‐induced liver and kidney damage. Overall, the findings suggest that the nanoliposomal delivery of GLE significantly boosts its therapeutic efficacy by reinforcing the body's natural antioxidant defenses, offering a more effective strategy for counteracting drug‐induced oxidative damage and preserving organ function.

### 3.10 Histological Evaluation of GLE Samples in Liver Tissues

**FIGURE 9:**
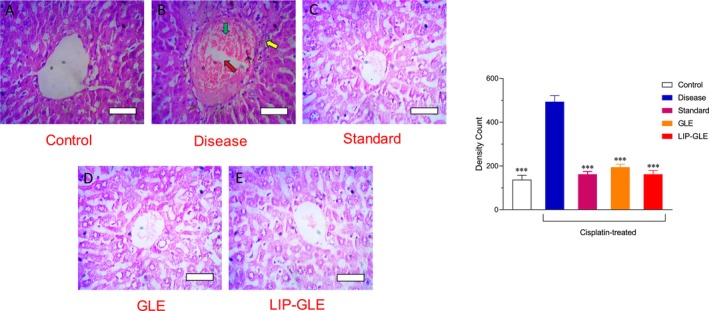
Histopathological examination of the effect LIP–GLE and GLE on rat liver induced by cisplatin using Hematoxylin and Eosin (H & E) staining. (A) Rats treated with saline, (B) cisplatin‐treated rats, (C) cisplatin‐treated rats with silymarin, (D) cisplatin‐treated rats with GLE, and (E) cisplatin‐treated rats with LIP–GLE; H & E of the liver of the diseased rats (cisplatin) showed obstruction of the central vein suggesting sclerosis (green arrow), cellular degranulation (yellow arrow), and the bile duct is inflamed (red arrow) and obstructed. ***p ≤ 0.001 versus control or disease or treatment groups (*n* = 6).

**FIGURE 10:**
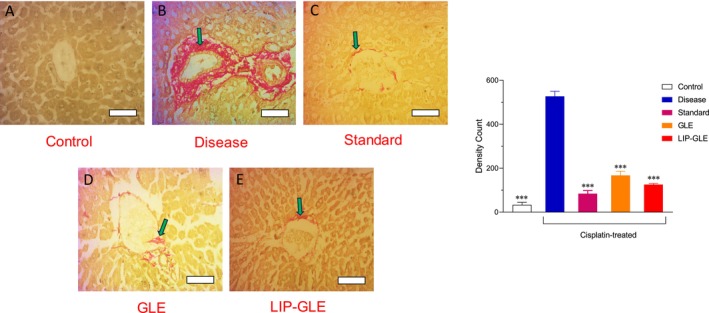
Histopathological examination of the effect of LIP–GLE and GLE on rat liver induced by cisplatin using Sirius Red staining. (A) Rats treated with saline, (B) cisplatin‐treated rats, (C) cisplatin‐treated rats with silymarin, (D) cisplatin‐treated rats with GLE, and (E) cisplatin‐treated rats with LIP–GLE; cisplatin intoxication showed accumulation of collagen mass in the liver (green arrow). ***p ≤ 0.001 versus control or disease or treatment groups (*n* = 6).

Histopathological evaluation of liver tissues stained with H&E and Sirius Red provided critical insight into the hepatoprotective potential of GLE samples, illustrated in Figures 9 and **10**. The control group displayed typical hepatic architecture, characterized by well‐preserved hepatocyte cords, intact central veins, and minimal inflammatory infiltration (Figure 9A), with Sirius Red staining revealing negligible collagen deposition, indicating healthy liver function and minimal fibrosis (Figure 10A). In contrast, the disease group treated with cisplatin alone (Figure 9B) showed severe hepatic damage, evidenced by pronounced ballooning degeneration of hepatocytes, sinusoidal dilation, congestion, and marked inflammatory cell infiltration. Sirius Red staining in this group revealed extensive collagen accumulation around the central vein and portal triad, suggesting substantial fibrosis and extracellular matrix deposition due to oxidative and inflammatory insults (Figure 10B). The standard treatment group with silymarin (Figure 9C) showed considerable improvement in liver architecture, with moderate reduction in hepatocyte damage and inflammatory changes, along with visibly decreased collagen deposition. However, some fibrotic patches remained (Figure 10C). The group treated with GLE (Figure 9D) exhibited partial protection, with moderate hepatocyte preservation, reduced inflammation, and mild fibrosis compared to the disease group, indicating the hepatoprotective role of GLE, though limited by its lower bioavailability. Significantly, the LIP–GLE group (Figure 9E) demonstrated the most notable histological recovery, with nearly restored liver architecture resembling the control group's. Hepatocytes appeared well‐organized with minimal inflammatory infiltration and no significant vascular congestion. Sirius Red staining confirmed a substantial reduction in collagen deposition, indicating a reversal of fibrosis and effective mitigation of tissue remodeling (Figure 10E). In the liver, inflammatory cell infiltration density significantly increased (3.6‐fold) in the cisplatin control group compared to the control group, while collagen deposition area increased in the cisplatin group (15.8‐fold) compared to the control group (*p* < 0.05). Meanwhile, LIP–GLE markedly reduced inflammation (3.1‐fold) and fibrosis (4.2‐fold) compared to the cisplatin group. Thus, based on the qualitative and quantitative histopathological observations, the superior hepatoprotective effect of the LIP–GLE can be attributed to the restored hepatic architecture, minimal inflammation, and significantly reduced fibrotic changes, indicating an effective reversal of cisplatin‐induced hepatic injury (Aboraya et al. 2022). These histopathological findings correlate strongly with improved liver function biomarkers and oxidative stress markers in the LIP–GLE group, reinforcing the assumption that nanoliposomal formulation offers a promising therapeutic strategy to combat cisplatin‐induced hepatic injury through improved delivery and enhanced biological efficacy of its active compounds.

### 3.11 Histological Evaluation of GLE Samples in Kidney Tissues

**FIGURE 11:**
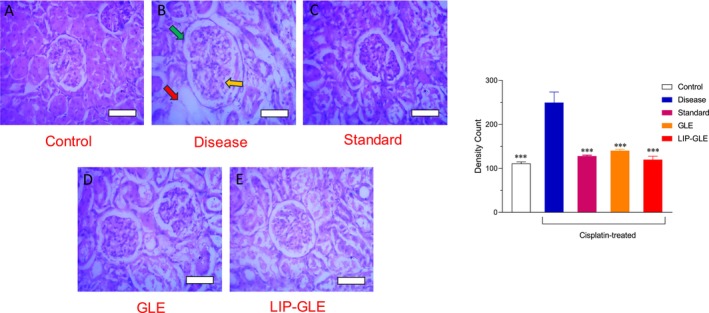
Histopathological examination of the effect LIP–GLE and GLE on rat kidney induced by cisplatin using hematoxylin and eosin staining. (A) Rats treated with saline, (B) cisplatin‐treated rats, (C) cisplatin‐treated rats with silymarin, (D) cisplatin‐treated rats with GLE, and (E) cisplatin‐treated rats with LIP–GLE; cisplatin‐challenged rats showed the size of the Bowman's capsule (green arrow), and the glomerulus showed hypercellularity (yellow arrow), presenting glomerulonephritis. Tubules showed distortion and hypertrophy (red arrow). ***p ≤ 0.001 versus control or disease or treatment groups (*n* = 6).

**FIGURE 12:**
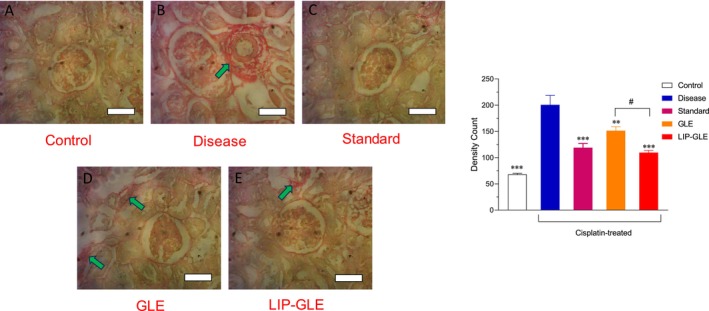
Histopathological examination of the effect LIP–GLE and GLE on rat kidney induced by cisplatin using Sirius Red staining. (A) Rats treated with saline, (B) cisplatin‐treated rats, (C) cisplatin‐treated rats with silymarin, (D) cisplatin‐treated rats with GLE, and (E) cisplatin‐treated rats with LIP–GLE; cisplatin intoxication showed accumulation of collagen tissues in the kidney (green arrow). **p < 0.01; ***p < 0.001; with respect to cisplatin‐treated rats; #*p* < 0.05, GLE vs. LIP–GLE. Data represents Mean ± SEM; (*n* = 6).

Histopathological analysis of kidney tissues using H&E and Sirius Red staining provided comprehensive insights into the nephroprotective effects of GLE samples in cisplatin‐induced nephrotoxicity, as depicted in Figures 11 and **12**. The control group (Figure 11A) exhibited normal renal histology with intact glomeruli, well‐organized proximal and distal tubules, and an absence of inflammatory infiltrates or fibrotic changes, confirming healthy renal architecture using H&E staining. Conversely, the disease group treated with cisplatin alone (Figure 11B) showed severe structural disruption, including tubular necrosis, glomerular atrophy, cast formation, marked interstitial inflammation, and vascular congestion. Sirius Red staining in this group revealed intense collagen accumulation, especially in the interstitial and peritubular areas, indicating significant fibrosis and extracellular matrix deposition as a result of oxidative stress and inflammatory responses (Figure 12B). The standard silymarin‐treated group (Figure 11C) demonstrated partial restoration of renal structure, with a noticeable reduction in tubular damage and inflammatory infiltration, and a moderate decrease in collagen deposition, reflecting its known nephroprotective and antifibrotic properties. The group treated with GLE (Figure 11D) exhibited moderate protective effects, showing some improvement in tubular morphology and a reduction in fibrosis compared to the disease group, though not as pronounced as the silymarin group, likely due to the limited solubility and bioavailability of the free extract. The LIP–GLE (Figure 11E) notably displayed substantial histological improvement, with preserved glomerular and tubular structures, minimal cellular infiltration, and negligible vascular congestion. Sirius Red staining revealed a marked reduction in fibrotic regions, indicating minimal collagen deposition and an effective attenuation of cisplatin‐induced renal fibrosis (Figure 12E). Inflammatory cell infiltration density was elevated by approximately 2.2‐fold in the cisplatin group compared to the control, while collagen deposition increased by about 3.0‐fold (*p* < 0.001). Treatment with LIP–GLE markedly attenuated both inflammation (2.1‐fold reduction) and fibrosis (1.8‐fold reduction) relative to the cisplatin group. The pronounced nephroprotective effect observed in the LIP–GLE underscores the advantages of nanoliposomal encapsulation, which enhances the solubility, stability, and cellular uptake of the extract's bioactive compounds, resulting in superior antioxidant, anti‐inflammatory, and antifibrotic effects (Halder et al. 2024). These findings align with biochemical assessments, where the LIP–GLE demonstrated significant improvement in kidney function markers, reduced oxidative stress, and enhanced antioxidant enzyme activity. Therefore, the histopathological restoration observed in the LIP–GLE underscores its significant renoprotective capabilities against cisplatin‐induced damage, reinforcing the efficacy of the nanoliposomal approach in mitigating renal injury.

## 4 Conclusions

LIP–GLE demonstrated strong protective effects against cisplatin‐induced liver and kidney toxicity in rats. The liposomal formulation proved more effective than GLE, as shown by improved biomarkers of liver and kidney function, increased antioxidant enzyme activities, and reduced oxidative stress markers. Histopathological evaluations confirmed liver and kidney structure preservation, with minimal fibrosis and inflammation observed in the LIP–GLE group. These improved therapeutic outcomes are likely due to the enhanced dispersibility, solubility, and bioavailability of the extract and the targeted delivery of its bioactive components through liposomal encapsulation. Thus, the findings suggest that LIP–GLE could be a promising therapeutic strategy for reducing cisplatin‐induced damage to the liver and kidneys, offering a novel and effective way to boost the nutraceutical benefits of GLE.

## Author Contributions

**Leon Bhowmik:** formal analysis (equal), investigation (equal), methodology (equal), writing – original draft (equal). **Ashikur Rahman:** formal analysis (equal), investigation (equal), methodology (equal), writing – original draft (equal). **Madhobi Karmakar:** data curation (equal), formal analysis (equal), investigation (equal), methodology (equal). **S. M. M. Sharif Nowaz Antu:** data curation (equal), visualization (equal), writing – review and editing (equal). **Zahidul Islam Zahid:** data curation (equal), visualization (equal), writing – review and editing (equal). **Madhabi Lata Shuma:** formal analysis (equal), investigation (equal), supervision (equal), visualization (equal), writing – original draft (equal), writing – review and editing (equal). **Shazid Md. Sharker:** conceptualization (equal), investigation (equal), project administration (equal), visualization (equal), writing – review and editing (equal). **Hasan Mahmud Reza:** resources (equal), supervision (equal), visualization (equal), writing – review and editing (equal). **Shimul Halder:** conceptualization (equal), investigation (equal), project administration (equal), resources (equal), software (equal), supervision (equal), validation (equal), visualization (equal), writing – original draft (equal), writing – review and editing (equal). **Manik Chandra Shill:** conceptualization (equal), funding acquisition (equal), project administration (equal), resources (equal), supervision (equal), visualization (equal), writing – review and editing (equal).

## Conflicts of Interest

The authors declare no conflicts of interest.

## Data Availability

All data supporting the findings of this study are available within the manuscript.
