# Correction to “Nanoliposomal Formulation of *Gynura procumbens* Leaf Extract Potentiates Hepatorenal Protection in Cisplatin‐Induced Rats”

**DOI:** 10.1002/fsn3.71373

**Published:** 2025-12-17

Bhowmik, L., A. Rahman, M. Karmakar, et al. 2025. “Nanoliposomal Formulation of Gynura procumbens Leaf Extract Potentiates Hepatorenal Protection in Cisplatin‐Induced Rats.” *Food Science & Nutrition* 13, no. 10: e71035. https://doi.org/10.1002/fsn3.71035.

In the originally published version, the surname for Ashikur Rahman was incorrectly spelled as Ashikur Rahaman. The online version has been corrected accordingly.

We apologize for this error.

